# Cube-Rhombellane Related Structures: A Drug Perspective

**DOI:** 10.3390/molecules23102533

**Published:** 2018-10-04

**Authors:** Mircea Vasile Diudea, Claudiu Nicolae Lungu, Csaba Levente Nagy

**Affiliations:** Department of Chemistry, Faculty of Chemistry and Chemical Engineering, Babes-Bolyai University, Arany J. street 11, Cluj 400028, Romania; lunguclaudiu5555@gmail.com (C.N.L.); nc35chem@gmail.com (C.L.N.)

**Keywords:** rhombellane, topology, DFT, pharmacological properties

## Abstract

Rhombellanes represent a new class of structures, of which homeomorphs may be synthesized as real molecules. Cube-rhombellane is a double-shell structure, with vertices of degree 3 and 6, respectively. Several hypothetical structures/molecules were proposed and computed using molecular graph theory and coordination chemistry principles. Some geometries were optimized at the B3LYP/6-31G (d, *p*) level of theory, followed by harmonic vibrational frequency analysis at the same level of theory, single point data were collected in view of molecular stability evaluation. Some of the bioactive functionalized structures were also proposed and explored by molecular mechanics (MM)-based conformational analysis, to check their internal mobility. Drug-like properties of the proposed molecular structures were compared with some existing nano-molecules (fullerenes, nanotubes). ADME and other physico-chemical characteristics were computed using commercial software. Substructures of the proposed molecules, useful in a future synthesis, were provided by retro combinatorial synthesis (RECAP). Computational results obtained are promising regarding ADME properties, drug-likeness and nano-properties.

## 1. Introduction

Crystal engineering and self-assembly have recently promoted new classes of finite and periodic crystalline solids, with promising applications in material science and biosciences [1,2,3,4,5,6,7]. The self-assembly principles have been applied to synthesize nano-sized spheroid architectures, based on Platonic and Archimedean solids. A variety of appropriate (as angles) ligands have been used in such syntheses to provide homeomorphs (see below) of the above solids. As metals to join the ligands, the most used was Pd^2+^ [3,4,5,6], then Zn^2+^ [7] or Cu^2+^ [8]. There are some polyhedra (like cuboctahedron, rhombicuboctahedron, etc.), eventually called “faceted polyhedra” [9], containing both open and closed faces; for the synthesis of such structures, self-assembly of triangles and squares is needed [7]. The procedure of structure self-assembly was termed “molecular meccano” [10]. The single-shell spherical structures, possessing large hollows, could be functionalized, both endo- and/or exohedrally. A self-assembled double-shell structure, a sphere-in-sphere, molecular “Russian doll”, was also reported [11].

Rhombellanes are structures with all strong rings being rhombs/squares, some of them forming local propellane substructures; they have been proposed by Diudea in 2017 [12]. Propellane is an organic molecule, first synthesized in 1982 [13]; by IUPAC nomenclature, it is named tricyclo[1.1.1.01,3]pentane, a hydrocarbon with formula C_5_H_6_. The reduced form of propellane, C_5_H_8_, eventually named bicyclo[1.1.1]pentane, has only rhomb/square rings; it can be represented as K_2.3_- the complete bipartite graph. The two bridge carbon atoms can be functionalized, e.g., by bromine or COOH, or even by repeating the K_2.3_ motif, as in the polymer called staffane [14].

A rhombellane was defined by Diudea [15,16,17] as the structure having: (a) All strong rings are squares/rhombs; (b) Vertex classes consist of all non-connected vertices; (c) Omega polynomial has a single term: 1X^|E|^; (d) Line-graph of the parent graph has a Hamiltonian circuit; (e) It contains at least one K_2.3_ subgraph.

**Figure 1:**
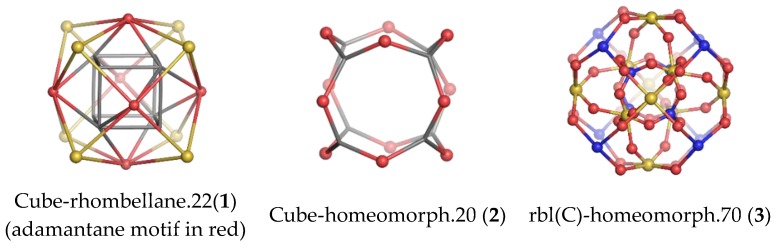
Cube-rhombellane and its related homeomorphs.

Construction of the cube-rhombellane (**1**) is illustrated in Figure 1, left. Each square face forms a K_2.3_ rhombellane by joining the opposite corners with homeomorphic diagonals; these diagonals are joint together in an adamantane motif (in red); K_2.3_ and adamantane are both “tiles” [18], not polyhedra [19], cf. Steinitz theorem [20].

A homeomorph of a graph contains on each parent edge one (or more) point(s) of degree two, e.g., the cube homeomorph **2** in Figure 1 (middle). The structure (**3**) (Figure 1, right), which is the homeomorph of (**1**) has seventy points/atoms; the vertex connectivity in (**3**) is 6; 3 and 2, respectively.

**Figure 2:**
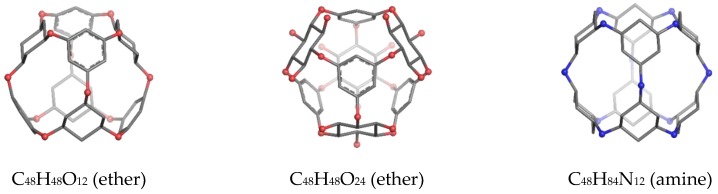
Cube-rbl-core(**4**) structures.

To synthesize **3** as a molecule, one may start from 1,2,3,4,5,6-Hexahydroxy-cyclohexane, that may form an ether **4** (also can be an amine) (Figure 2), which is a (hyper) homeomorph of the cube (**2**) and the “core” of rbl(C)-homeomorph **3**; the vertices of connectivity 6 will be just the hexahydroxy-cyclohexane while the three-connected points may be 1,3,5-trihydroxycyclohexane or its derivatives (e.g. hexahydroxycyclohexane, 1,3,5-trihydroxybenzene, etc.) [21].

There is a trend in supramolecular chemistry in the synthesis of Platonic or Archimedean polyhedral clusters by chemical fragments, eventually with metallic ions complexing suitable ligands. In this respect, homeomorphs of the cube, of various chemical composition, have been already synthesized [6,8,22,23]. For cubane and related structures see refs. [24,25,26].

**Figure 3:**
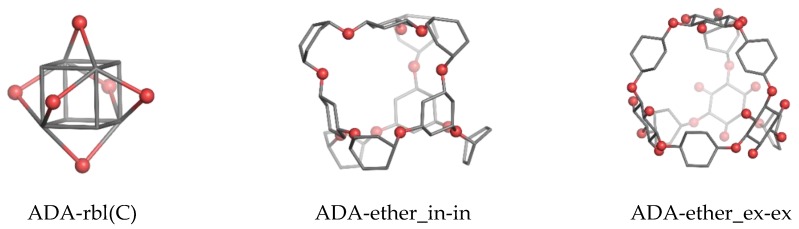
Adamantane motif in cube rhombellanes.

**Figure 4:**
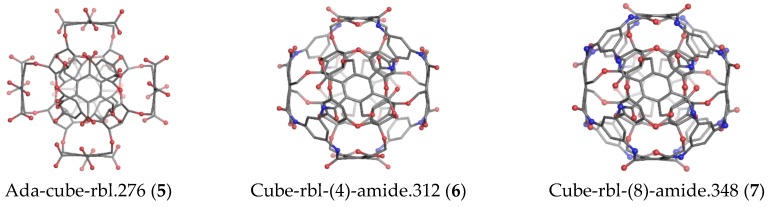
Cube-rhombellanes.

Cube-rhombellane **1** comprises an adamantane motif (Figure 3, left) that, in homeomorphs, becomes a hyper-adamantane, e.g., an ADA-ether (Figure 3, middle and right).An ADA-cube-rbl **5** (Figure 4, left) is a functionalized hyper-adamantane, consisting of a rbl-core bound by six units (one for each face of the cube) derived from benzene-1,2,4,5-tetracarboxylic acid. Then, by completing the second/external shell (by adding four or eight *tri*-connected units), double-shell Cube-rbl-amides **6**/**7** (Figure 4, middle and right) are obtained [21]; the envelope of **7** (or the second, external shell) will be counted as **8**.

All the molecular structures discussed herein are hypothetical molecules, not yet synthesized. 

The aim of this paper is to introduce the cube-rhombellanes, belonging to the newly introduced structural class, as possible biologically active compound candidates.

## 2. Results

The structures discussed in the following are listed in Table 1. The cube-rhombellane homeomorphs are named as: Cube-rbl-(a.b.c.d)-amide/ester, where the a.b.c.d bits represent: hexa-connected unit, *tri*-connected unit, connection to the second shell and the *tri*-connected units in the second/external shell, respectively. Structures were designed and computed by our original Nano Studio software [27]. 

### 2.1. DFT Results

**Figure 5:**
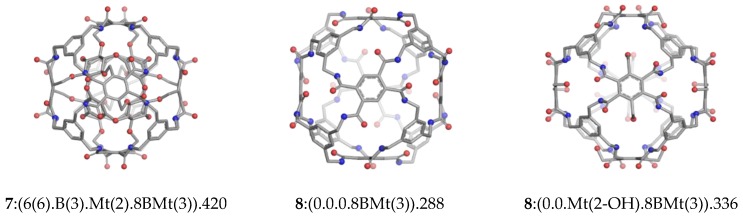
DFT optimized Cube-rhombellane structures: Cube-rbl-amide.420 (Tb2(1)) and its envelopes on 288 (Tb2(2)) and 336 (Tb2(3)) atoms; for structural specification see Table 1.

Cube-rbl-amide, **7**:(6(6).B(3).Mt(2).8BMt(3)).420, (Tb2(1); Tb2 = Table 2 – Figure 5, left), a double shell cluster with eight three-connected units in the second shell, has a tetrahedral *T* symmetry and is built from two larger fragments: the outer layer **8**:(0.0.0.8BMt(3)).288 (Tb2(2), Figure 5, middle) and the encapsulated core cluster **4**:(6(6).B(3).0.0)).120, (Tb2(4)). The two fragments are bounded together by twelve -CH_2_-O- linkers (see the bit Mt(2)) which connect the aromatic rings from the outer shell with the cyclohexane carbon atoms from the inner structure. Each cyclohexane ring is linked to three distinct benzene rings in the outer shell.

The core **4**:(6(3).B(3).0.0).108, (Tb2(5)) has a tetrahedral *T_d_* arrangement of four aromatic and four cyclohexane rings; if all eight units are identical, as in **4**:(6(3).6(3).0.0).132 (Tb2(6)), the symmetry of the structure is *O_h_*. When three hydroxyl groups are attached to each cyclohexane ring in equatorial positions, as in **4**:(6(6).B(3).0.0).120, (Tb2(4)), during geometry optimization the symmetry is lowered to *T*, attributed to the orientation of the hydrogen atoms in the hydroxyl groups and a small distortion in the carbon framework.

A similar structural deviation can be observed when attaching -CH_2_OH groups to the benzene rings aligned on the *C*_2_ symmetry axe in the envelope **8**:(0.0.0.8BMt(3)).288, (Tb2(2)). In the cluster **8**:(0.0.Mt(2−OH).8BMt(3)).336, (Tb2(3), Figure 5, right), these aromatic rings have a different orientation, being rotated by nearly 60° with respect to the opposite one lying on the same *C*_2_ rotational axe.

There are two types of aromatic rings in the envelopes **8**:(0.0.0.8BMt(3)).288 and **8**:(0.0.Mt(2−OH).BMt(3)).336 (one three−connected and the other four connected). These fragments have been optimized as molecules (Tb2(10) and Tb2(11), respectively). Their symmetry corresponds to the symmetry axes along which they are aligned.

Full geometry optimizations were performed using B3LYP/6−31G (d, *p*) on structures listed in Table 2. Computational results confirm that the investigated compounds have a good stability, attributed on the rather large HOMO−LUMO energy gaps. The binding energy, expressed as the difference between the total energy of the structure and the energies of the constituent atoms, shows that all the compounds are energetically feasible. Cluster **7**:(6(6).B(3).Mt(2).8BMt(3)).420, (Tb2(1)), with eight three−connected units in the second shell, has the largest energy gap (4.390 eV) compared to the molecules with incomplete second shell (Tb2(7):4.020 eV; Tb2(8): 3.692 eV; Tb2(9): 4.276 eV) – for their complete name see Table 1. A similar trend can be seen for their binding energy E_bind/N_ (Table 2).

### 2.2. Bioactivity Evaluation

Rhombellanic structures discussed in this section are also listed in Table 1.

#### 2.2.1. MM Computations

MM−energies [29] for the structures Tb3(1 to 3) were minimized (the shaded values—in kcal/mol—Table 3). The corresponding MM−energies for the reference fullerenes C_60_, C_70_ and nanotube (5,5), are: 2354.76; 1980.58; 2015.98, respectively. Only the structure Ada−C−rbl.276 (**1**) was also optimized at the B3LYP level of theory (Table 2—entry 9). The compounds Tb3(1 to 3) were further computed in order to assess their drug-like properties and consecutively their bioactive conformations.

#### 2.2.2. Globularity

**Figure 6:**
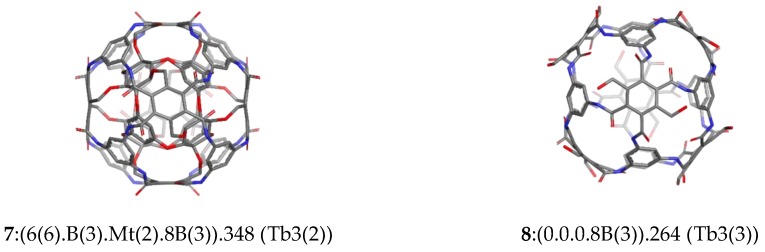
Cube rhombellane structures (Table 3).

**Figure 7:**
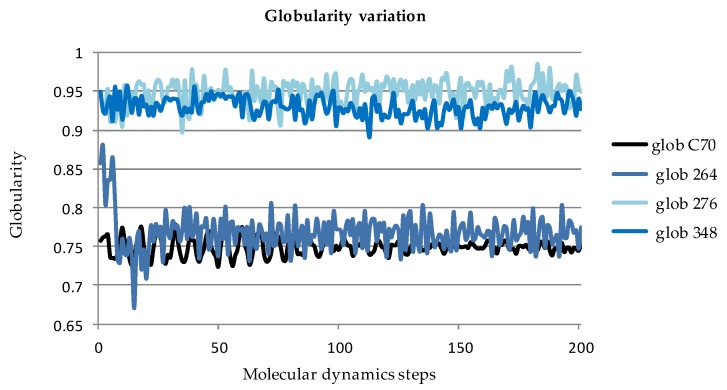
Computed globularity for some rhombellane related structures and fullerene C_70_ (see Supplementary Material S1).

Globularity [30] of rhombellanes, computed in a molecular dynamics (MD)-related manner, is superior to that of fullerene C_70_. The rhombellaneC_rbl.348 (Tb3(2), Figure 6) has computationally a globularity close to 1 – globularity of a perfect sphere (see Figure 7).

#### 2.2.3. ADME Properties Evaluation

Values of ADME [31,32] properties are listed in Table 4. Disposition of a pharmaceutical compound in organism is regarded as a function of compound lipophilic/hydrophilic properties, binding to serum proteins and permeability among cellular membranes. Env_264 (Tb3(3)) shows close to nanotube (5,5), the polarizability QPpolrz and decahexane gas partition coefficient QPlogPC16 values; the other investigated parameters are less optimistic in terms of drug likeness when compared to nanotubes and fullerenes C_70_ and C_60_.

When evaluating membrane permeability, rhombellanes show poor CNS permeability; BBB, i.e., blood brain barrier, (see QPPMDCK parameter—Table 5 or CNS values—Table 6) is not crossed by rhombellanes in contrast with the nanotubes and fullerenes, which show high penetration coefficient values. An exception is remarked for rhombellanes, particularly for C_rbl_348(Tb3(2)), which computationally express good skin permeability (parameter QPlogKp—Table 5).

Log *S*, the aqueous solubility of a compound, affects its absorption and distribution. A low solubility is correlated with modes absorption. 80% of the drugs on the market have log values greater than (−)4. In comparison with nanotubules and fullerenes some rhombellanes have decent solubilities ((−)6.95;(−)9.82).

In contrast to fullerenes and nanotubules, which are highly lipophilic (e.g., log *P* = 57 for nanotube 3,3), rhombellanes are hydrophilic (log *P* for the rhombellane C_rbl_348 (Tb3(2)) = (−)22.15) (see Supplementary Materials).

Orally, there is no potential absorption for rhombellanes (Table 6). Drug-like properties of rhombellanes are close to those of fullerenes and nanotubules (having only one extra violation of Lipinski‘s Rule of five [33] and Rule of three [34], respectively—Table 6).

A set of descriptors were computed (see Supplementary Materials—S2). The number of aromatic atoms is increased in rhombellanes in comparison with fullerenes and nanotubules and consecutively the atomic contribution to log *P*.

Flexibility of rhombellanes is about 30 times bigger than that of C_70_, according to Kier flexibility index [35]. Total hydrophobic van der Walls area and total positive van der Walls areas reflect the same properties. (see Supplementary Material S2).

**Figure 8:**
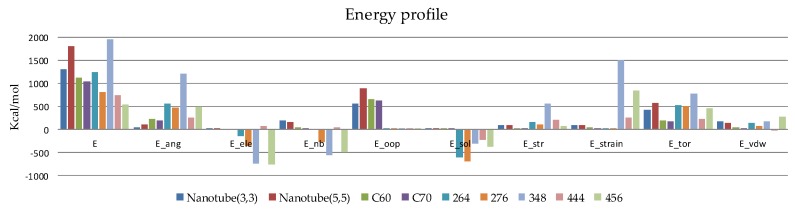
The energy profile (in kcal/mol) of the compounds listed in Table 3. Used abbreviations: *E*—potential energy; *E_ang*—angle between potential energy; *E_ele*—electrostatic component of potential energy; *E_oop*—out of plane potential energy; *E_sol*—solvation energy; *E_str*—bond stretch potential energy; *E_strain*—local strain energy; *E_tor*—torsional potential energy; *E_vdw*—van der Waals component of potential energy.

Rhombellanes carry more chemical information than fullerenes and nanotubules, when considering connectivity−based descriptors. Energy profiles (Figure 8), calculated using a MM2 force field [29] (the energy descriptors are calculated using the potential energy (in kcal/mol) from the stored 3D conformations) vary according to the same dynamics and confirm their internal mobility, higher than of the reference nano−molecules (fullerenes and nanotubes—see Table 3).

#### 2.2.4. RECAP Analysis 

For the identification of the molecular building blocks of rhombellane derivatives listed in Table 3, an analysis was performed using the Retrosynthetic Combinatorial Analysis Procedure (RECAP) method [36], the results being listed in Table 7.

Some RECAP novel molecules suggested for the synthesis using templates arising from RECAP analysis are shown in Table 8. (full output is given in Supplementary Material S3).

RECAP result structures, given in Table 7 and Table 8, show the chemical synthetic space of rhombellanes. Frequency of computed appearance reveals the following: 6−connected hexagonal rings (frequency (%): 1.252–8.493, Table 7, entries 1 to 6); 3−connected hexagonal rings (frequency (%): 2.877–14.358, Table 7, entries 7 to 10), with more plausible substructures those having a higher frequency. Substitution of aromatic core seems to be an important variable in the synthesis; stereochemistry of compounds has an important impact. Water molecules show a high frequency (94.106) suggesting its role as a reaction medium and possibly its formation as a by-product.

**Figure 9:**
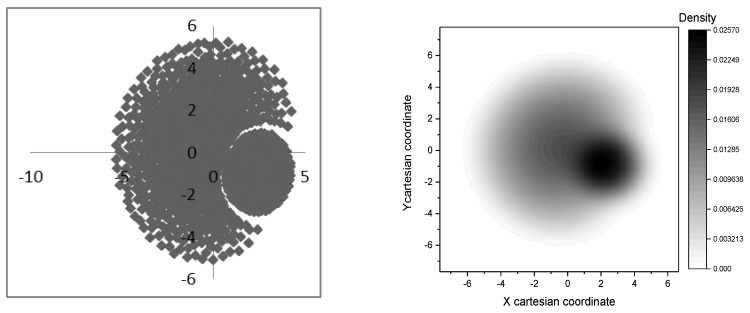
RECAP cluster analysis (left); cluster point to point representation (cluster density – right).

When analyzing the entire RECAP results, including all compounds (over 1000 results—see Supplementary Material S3), two major clusters were identified (Figure 9). Density analysis also demonstrates clusters identity. Among the novel molecules suggested synthesis using templates arising from RECAP analysis some structures, collected in Table 8, show ether constructions, possibly useful in a real synthesis.

## 3. Methods

Rhombellanes are constructed by the “rhombellation” procedure; it starts with diagonalizing each face of an all−rhomb map Rh_0_ by a joint point (called “rbl−point”); then, add new vertices opposite to the parent vertices and join each of them with the rbl−vertices lying in the proximity of each parent vertex, thus local Rh−cells being formed. The process can continue, considering the envelope Rh_n_ as “Rh_0_” for Rh_n+1_, in this way shell by shell being added to the precedent structure. Since the two diagonals of a rhomb may be topologically different, each generation may consist of two isomers.

Density functional theory (DFT) was used to investigate the stability of a series of rhombellanes (Table 2). Geometry optimizations was performed at the B3LYP/6−31G (d, *p*) level of theory using tight convergence criteria as implemented in the Gaussian 09 suite [28]. Harmonic vibrational frequencies obtained at the same level of theory confirmed that a true stationary point has been obtained. The obtained computational DFT results are collected in Table 2: the point group symmetry (PG), HOMO−LUMO energy gap (E_gap_), binding energy (E_bind_), binding energy per number of heavy atoms (E_bind/N_), and the total energy (E_tot_). DFT optimized Cartesian coordinates are given in Supplementary Material S4.

To obtain the ground state geometry of structure Tb2(11), multiple starting geometries were considered. The lowest energy configuration was the one where the hydroxyl groups are located on the same side of the benzene ring, while all carbonyl oxygen atoms are located on the opposite side. This arrangement is in agreement with the fragments observed in the double−shell cluster **7**:(6(6).B(3).Mt(2).8BMt(3)).420 (Tb2(1)).

Molecular mechanics (MM) were utilized in order to explore the internal molecular mobility, accessed in the potential bioactivity study of these compounds. MMFF94 force field [29] was used to sample for 10 conformation poses of molecular structures Tb3(1 to 5). Steric energy of each pose (kcal/mol) was computed (Table 3). Each structure was minimized for maximum of 100 times, 10 conformations were sampled and conformational energy for each sample computed (kcal/mol).

Globularity (the state of being globular [30]) of rhombellanes was computed in comparison with that of fullerene C_70_. Globularity correlates with bioactivity of molecules or with manifestation of bioactivity (i.e., bioactive molecules are globular). Results were interpreted according to globularity descriptor definition (i.e., a sphere shape molecule has globularity equal to 1). MD was used to explore globularity. The descriptor was computed for 200 conformations corresponding to 200 MD steps computed for a target temperature of 310.15K, uth a step interval of 1fs, frame interval of 1fs, and a heating/cooling rate of 1 kcal/atom/ps. For each structure the computation was terminated after 200 steps.

ADME properties evaluation was performed using QikProp [37] module included in Schrodinger software. A series of descriptors related to bioactivity and drug-ability of these compounds were computed (see supplemental material S2 and refs. [38,39,40]). Also, a comparative energy profile was calculated using the same module.

Retrosynthetic Combinatorial Analysis (RECAP) [41] was performed using a Knime workflow (RetroPath2.0); the analysis of structures revealed 10 molecular motives, utilizable as starting points for further fusion (Table 8).

RECAP computes a molecule by breaking certain bonds, estimated to be those that can be reformed by common reliable chemistry. Each resulting fragment is assigned a unique extended SMILES name that retains the chemical context of the broken bond. By applying this fragmentation methodology to a collection of compounds, statistics on individual fragments can be gathered and the fragments subsequently recombined in the “synthesis” procedure to generate new molecules.

RECAP resulted molecules were computed as clusters in order to identify their dispersion and correlation to chemical space. To make a cluster representation, the density analysis of resulted clusters was performed. RECAP analysis was used to generate novel molecules that can be, eventually, utilized in rhombellane synthesis.

## 4. Discussions

An active compound is a collection of molecular fragments according to fragment−based drug design principles. These fragments possess a specific three−dimensional arrangement that defines the whole properties of the compound: geometric, steric, conformational, topological, electronic and physicochemical properties define key aspects of bioactivity. By the medicinal chemistry point of view these properties define the therapeutic, metabolic and toxic properties.

Physicochemical properties are crucial to the pharmaceutical and pharmacokinetic phases of drug action; the pharmacodynamics are less discussed here and will be approached in another paper concerning these compounds. 

Electronic properties reflect electron distribution within the drug molecule and determine the nature of the precise binding interaction between the drug and its receptor through hydrogen bonding and other interactions. On this subject computed conformational energies of rhombellanes take positive values, whereas rhombellanes envelopes possess negative energies (Table 3). All 10 distinct conformational samples for each structure present alternate conformational energies suggesting an encouraging conformational mobility specific for drug carriers and bio−nano−devices. Their energy profile, when compared to each other, is balanced, presenting the same proportional energy variation (Figure 8).

Geometric, steric, and topological (globularity included) properties describe the structural arrangement of the atoms within the drug molecule and influence the interaction with the target. 

Physicochemical properties reflect the solubility and absorption characteristics (in aqueous and lipid environments) of the drug and its ability to cross barriers, such as the blood–brain barrier, on its way toward the receptor. Thus, a highly significant property of drug molecules is their solubility (both in aqueous *and* non−aqueous environments), because only in solution can they interact with the cellular and subcellular structures that carry drug receptors, thus triggering pharmacological reactions. Partition coefficients are extremely important when understanding the properties of drug molecules. Most successful drugs exhibit solubility to some extent both in water and lipid environments; however, there are a few examples in which solubility in only one of these phases correlates with pharmacological activity.

Solubility is a function of many molecular parameters. Ionization, molecular steric structure, size, and electronic structure (by heteroatom presence), all influence the basic interactions between a solvent and the solute. Physicochemical properties are important in determining the ability of a drug molecule to survive the pharmacokinetic phase and to reach the region of the receptor−important both in bioactive molecules and in drug transporters. In this regard, rhombellanes present absorption properties comparable with carbon nanostructures. CNS penetration is less favorable in comparison to nanotubes and fullerenes.

The correct geometry, conformation, and stereochemistry of a molecule enable it to gain access to the receptor microenvironment [42,43]. However, it is the electronic molecular structure that finally decides the electrostatic, hydrogen bonding, and other drug–receptor binding interactions to actually occur.

The effect of electron distribution in organic molecules is manifested directly (short range) or indirectly (long range). Inductive forces, as van der Waals bonds or dipole−dipole interactions, are the result of polarization or polarizability—the permanent or induced distortions of the electron distribution within a molecule. These forces are especially important in studies of quantitative structure–activity relationships (QSAR) because the electronic effect of a substituent can, by resonance or an inductive or field effect, change the stereo−electronic properties of a molecule and thus influence its biological activity.

Statistical analysis is used frequently in bioinformatics. Density−based clustering defines clusters as areas of higher density than the remainder of the data set. In this study, OPTICS was used for clustering the RECAP results; OPTICS [44] is a generalization of DBSCAN which avoids the need to choose an appropriate value for the range parameter epsilon and produces a hierarchical result related to that of linkage clustering.

Based on this findings future developments of these compounds will be carried on. Using reverse synthesis techniques a chemical synthesis will be tested. Furthermore optimized compounds refined regarding geometry and outer surface and inner core—shell and envelope—are intended to be tested against biological targets.

## 5. Conclusions

Rhombellanes represent a new class of hypothetical structures, of which homeomorphs may be synthesized as real molecules. Cube−rhombellane, in the first step of rhombellation operation, is a double−shell structure showing vertices of degree 3 and 6 respectively, which may be realized by means of cyclohexane (and/or benzene) derivatives. Some suggestions of molecular realization of cube−rhombellane as functionalized structures (ether core, ester or amide envelope) were given. Topology of the discussed structures was detailed. Quantum calculations supported the herein hypothesis: both substructures (at every level of complexity) and the whole double−shell molecules show negative binding energy and a rather high energy gap, proving that all the compounds are energetically feasible in the hope of a real synthesis.

Rhombellanes seem to be appropriate and worthwhile molecules for medicinal chemistry. This new class of compounds may be an alternative to classical nano−structures regarding carrier, drug like properties and use for creating bio−nano devices. In contrast to fullerenes and nanotubules, where most of functionalization (and derivatives) occur symmetrically, rhombellanes present a variety of distinct derivatization sites thus making them easier to be chemically manipulated. As drug carriers, rhombellanes show decent membrane-passing properties. In close relation with fullerenes and nanotubules, our compounds present “*per se*” drug-like properties which could be further developed to access a novel chemical space.

## Figures and Tables

**Table 1:** Cube-rhombellanes: structure; v = no. vertices/atoms; type.

Structure	v	C	H	N	O	Table	Type I	Type II
C-rbl	420	192	156	24	48	2(1)	Ether; Amide	**7**:(6(6).B(3).Mt(2).8BMt(3))
Env (420)	288	132	108	24	24	2(2)	Amide	**8**:(0.0.0.8BMt(3))
Env (420)	336	144	132	24	36	2(3)	Amide	**8**:(0.0. Mt(2-OH).8BMt(3))
Core	120	48	48	0	24	2(4)	Ether	**4**:(6(6).B(3).0.0)
Core	108	48	48	0	12	2(5)	Ether	**4**:(6(3).B(3).0.0)
Core	132	60	60	0	12	2(6)	Ether	**4**:(6(3).6(3).0.0)
C-rbl (4)	264	132	84	0	48	2(7)	Ether; Ester	**6**:((6(6).Mt(2).4B(3))
C-rbl (4)	300	144	84	0	72	2(8)	Ether; Ester	**6**:((6(6).Mt(2-COOH).4B(3))
Ada-C-rbl	276	120	84	0	72	2(9); 3(1)	Ether	**5**:(6(6).B(3).Mt(2).0)
C-rbl	348	168	108	24	48	3(2)	Ether; Amide	**7**:(6(6).B(3).Mt(2).8B(3))
Env (348)	264	120	84	24	36	3(3)	Amide	**8**:(0.0.0.8B(3))
C-rbl	444	192	180	24	48	3(4)	Ether; Amide	**7**:(6(6).6(3).Mt(2).8BMt(3))
C-rbl	456	156	192	24	84	3(5)	Ether; Amide	**7**:(6(6).6(6).Mt(2).8BMt(3))

Env = envelope; B = benzene; Mt = methylene; (*n*) = fragment connectivity.

**Table 2:** Computational results obtained at the B3LYP/6-31G (d, *p*) level of theory using the Gaussian 09 suite [28]: the point group symmetry (PG), HOMO-LUMO energy gap (E_gap_), binding energy (E_bind_), binding energy per number of heavy atoms (E_bind/N_), and the total energy (E_tot_).

	Formula	Total Atoms	PG	E_gap_ (eV)	E_bind_ (a.u.)	E_bind_/_N_ (kcal/mol)	E_tot_ (a.u.)
1	C_192_H_156_N_24_O_48_	420	*T*	4.390	−93.497	−222.236	−12334.939
2	C_132_H_108_N_24_O_24_	288	*T*	4.451	−65.811	−229.427	−8215.224
3	C_144_H_132_N_24_O_36_	336	*T*	4.965	−73.981	−227.568	−9589.441
4	C_48_H_48_O_24_	120	*T*	6.104	−24.528	−236.370	−3663.244
5	C_48_H_48_O_12_	108	*T_d_*	6.200	−22.601	−213.768	−2760.590
6	C_48_H_72_O_12_	132	*O_h_*	6.139	−24.906	−260.481	−2774.902
7	C_132_H_84_O_48_	264	*T*	4.020	−59.334	−206.849	−8690.700
8	C_144_H_84_O_72_	300	*T*	3.692	−67.266	−195.418	−10953.399
9	C_120_H_84_O_72_	276	*T*	4.276	−59.767	−195.335	−10039.276
10	C_12_H_15_N_3_O_3_	33	*C*_3_	6.282	−6.854	−238.962	−856.290
11	C_16_H_22_N_4_O_6_	48	*C*_2_	5.220	−9.666	−233.289	−1293.368

**Table 3:** MM conformational energies for the molecular structures Tb3(**1** to **5**) (kcal/mol).

	Ada−C−rbl.276 (1)	C_rbl.348 (2)	Env.264 (3)	C_rbl.444 (4)	C_rbl.456 (5)
1	412.152	1876.9	133.294	754.677	1334.91
2	411.509	1888.4	133.281	770.938	1275.35
3	407.547	1872.15	133.086	776.412	1301.15
4	445.824	1931.21	105.310	793.852	1354.78
5	387.843	1916.52	150.839	778.074	1305.25
6	392.251	1858.42	141.293	820.662	1303.09
7	413403	1866.64	136.255	774.167	1283.52
8	421.086	1857.53	108.876	779.648	1301.57
9	435.597	1907.43	125.237	821.927	1293.61
10	439.588	1867.08	155.056	816.659	1336.80

**Table 4:** Hydrophilic/lipophilic partition related descriptors: QPpolrz − predicted polarizability in Å3 n.v. (−)13.0−70.0; QPlogPC16−predicted hexadecane/gas partition coefficient n.v. (−)4−18; QPlogPoct−predicted octanol/gas partition coefficient n.v. (−)8.0−35.0; QPlogPw−predicted water/gas partition coefficient n.v. (−)4.0 −45.0; QPlogKhsa− prediction of binding to human serum albumin n.v. (−)1.5−1.5; QPlogPo/W−predicted octanol/water partition coefficient n.v. (−)2−6.5.

Molecule	QPpolrz	QPlogPC16	QPlogPoct	QPlogPw	QPlogKhsa	QPlogPo/w
Nanotube (5,5)	172.56	40.958	70.161	14.096	10.553	23.631
Nanotube (3,3)	102.772	25.308	41.65	6.671	5.881	13.667
C_70_	58.28	16.258	22.272	3.89	2.853	8.587
C_60_	50.017	14.398	18.716	3.444	2.292	7.599
C_rbl_456 (3(5)	196.72	59.101	191.881	189.665	−17.697	−26.217
C_rbl_444 (3(4))	192.178	54.083	166.025	152.579	−14.823	−18.859
C_rbl_348 (3(2))	180.498	46.315	169.989	151.045	−4.982	−11.232
Ada−C−rbl_276 (3(1))	155.706	58.948	155.222	120.044	−11.895	−5.558
Env_264 (3(3))	152.497	49.581	157.638	151.739	−12.271	−25.591

Regarding of binding to albumin (QPlogKhsa parameter—Table 4), nanotube (3,3) and C_70_, C_60_ show optimal albumin binding; rhombellanes do not reflect such a property.

**Table 5:** Membrane related properties: QPlogS− predicted aqueous solubility n.v.(−)6.5 −0.5; ClQPlogS−conformation−independent predicted aqueous solubility n.v. (−)6.5 −0.5; QPlogHERG − predicted IC50 value for blockage of HERG K+ channels n.v. concentration below (−)5; QPPCaco− predicted apparent Caco−2 cell permeability (in nm/sec). Caco−2 cells are a model for the gutblood barrier n.v. < 25por, >500 excellent; QPlogBB−predicted brain/blood partition coefficient n.v. (−)3−1.2; QPPMDCK− predicted apparent MDCK cell permeability (blood brain barrier − in nm/sec), n.v. < 25por, > 500 excellent; QPlogKp− predicted skin permeability n.v. (−)8−1.0.

Molecule	QPlogS	CIQPlogS	QPlogHERG	QPPCaco	QPlogBB	QPPMDCK	QPlogKp
Nanotube (5,5)	−55.716	−55.716	−11.271	9906.038	0.192	5899.293	3.712
Nanotube (3,3)	−32.425	−32.425	−6.455	9906.038	0.192	5899.293	1.726
C_70_	−17.329	−17.329	−4.863	9906.038	0.192	5899.293	0.714
C_60_	−14.534	−14.534	−4.634	9906.038	0.192	5899.293	0.517
C_rbl_456 (Tb3(5)	2	−6.18	29.537	0	−13.489	0.002	0.76
C_rbl_444 (Tb3(4))	2	−12.722	29.742	0	−12.202	0.007	0.52
C_rbl_348 (Tb3(2))	2	−17.995	16.156	0	−6.523	0.003	−6.921
Ada−C−rbl_276(Tb3(1))	2	−14.584	37.724	0	−24.685	0	−24.438
Env_264 (Tb3(3))	2	3.158	29.921	0	−11.775	0	−12.728

**Table 6:** Drug−like properties of rhombellane derivatives.

Molecule	CNS	Human Oral Absorption	Rule of Five Violation	Rule of Three Violation
Nanotube (5,5)	1	1	2	1
Nanotube (3,3)	1	1	2	1
C_70_	1	1	2	1
C_60_	1	1	2	1
C_rbl_456 (Tb3(5)	−2	1	3	2
C_rbl_444 (Tb3(4))	−2	1	3	2
C_rbl_348 (Tb3(2))	−2	1	3	2
Ada−C−rbl _276 (Tb3(1))	−2	1	3	2
Env_264 (Tb3(3))	−2	1	3	2

**Table 7:** RECAP analysis result: molecules represented as 2D formulas, molecular mass (M), and the corresponding frequency of appearance (%) in the analysis.

Structure	Frequency (%)	Structure	Frequency (%)
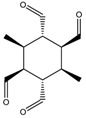 M = 31.295	1.212	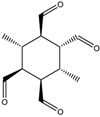 M = 30.820	1.212
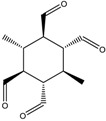 M = 29.809	1.212	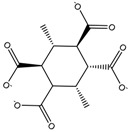 M = 251.659	3.773
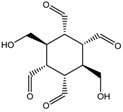 M = 31.034	4.692	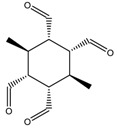 M = 30.612	8.493
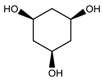 M = 10.646	2.877	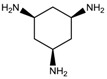 M =14.352	4.721
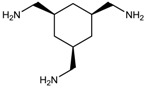 M =10.492	14.352	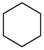 M =14.358	6.556

**Table 8:** Molecular fragments identified by RECAP novel molecules for further fusion, molecular mass (M) of the compounds are given below their formula.

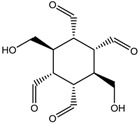	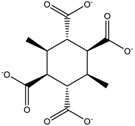	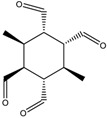
M =256.254	M = 284.22	M = 224.256
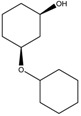	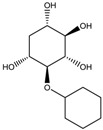	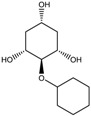
M = 198.306	M = 246.303	M = 230.304
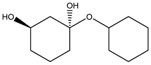	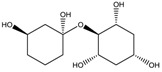	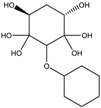
M = 214.305	M = 262.302	M = 278.301
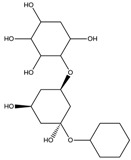	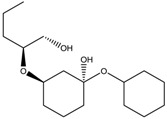	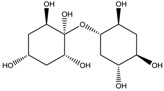
M = 376.446	M = 312.45	M = 294.3

## Supplementary Materials

The following are available online. S1: globularity, S2: descriptors, S3: RECAP results, S4: DFT coordinates.
